# Occurrence of the Middle East Respiratory Syndrome Coronavirus (MERS-CoV) across the Gulf Corporation Council countries: Four years update

**DOI:** 10.1371/journal.pone.0183850

**Published:** 2017-10-13

**Authors:** Mahmoud Aly, Mohamed Elrobh, Maha Alzayer, Sameera Aljuhani, Hanan Balkhy

**Affiliations:** 1 King Abdullah International Medical Research Center, Riyadh, Saudi Arabia; 2 King Saud bin Abdulaziz University for Health Sciences, Riyadh, Saudi Arabia; 3 Department of Biochemistry, College of Sciences, King Saud University Riyadh, Saudi Arabia; 4 King Abdulaziz Medical City, Riyadh, Saudi Arabia; Dasman Diabetes Institute, KUWAIT

## Abstract

The emergence of the Middle East Respiratory Syndrome Coronavirus (MERS-CoV) infections has become a global issue of dire concerns. MERS-CoV infections have been identified in many countries all over the world whereas high level occurrences have been documented in the Middle East and Korea. MERS-CoV is mainly spreading across the geographical region of the Middle East, especially in the Arabian Peninsula, while some imported sporadic cases were reported from the Europe, North America, Africa, and lately Asia. The prevalence of MERS-CoV infections across the Gulf Corporation Council (GCC) countries still remains unclear. Therefore, the objective of the current study was to report the prevalence of MERS-CoV in the GCC countries and to also elucidate on its demographics in the Arabian Peninsula. To date, the World Health Organization (WHO) has reported 1,797 laboratory-confirmed cases of MERS-CoV infection since June 2012, involving 687 deaths in 27 different countries worldwide. Within a time span of 4 years from June 2012 to July 2016, we collect samples form MERS-CoV infected individuals from National Guard Hospital, Riyadh, and Ministry of health Saudi Arabia and other GCC countries. Our data comprise a total of 1550 cases (67.1% male and 32.9% female). The age-specific prevalence and distribution of MERS-CoV was as follow: <20 yrs (36 cases: 3.28%), 20–39 yrs (331 cases: 30.15%), 40–59 yrs (314 cases: 28.60%), and the highest-risk elderly group aged ≥60 yrs (417 cases: 37.98%). The case distribution among GCC countries was as follows: Saudi Arabia (1441 cases: 93%), Kuwait (4 cases: 0.3%), Bahrain (1 case: 0.1%), Oman (8 cases: 0.5%), Qatar (16 cases: 1.0%), and United Arab Emirates (80 cases: 5.2%). Thus, MERS-CoV was found to be more prevalent in Saudi Arabia especially in Riyadh, where 756 cases (52.4%) were the worst hit area of the country identified, followed by the western region Makkah where 298 cases (20.6%) were recorded. This prevalence update indicates that the Arabian Peninsula, particularly Saudi Arabia, is the hardest hit region regarding the emerging MERS-CoV infections worldwide. GCC countries including Saudi Arabia now have the infrastructure in place that allows physicians and scientific community to identify and immediately respond to the potential risks posed by new outbreaks of MERS-CoV infections in the region. Given the continuum of emergence and the large magnitude of the disease in our region, more studies will be required to bolster capabilities for timely detection and effective control and prevention of MERS-CoV in our region.

## Background

The emergence of MERS-CoV dates back to July 2012 when an elderly patient of age 60 years died from an acute pneumonia in Saudi Arabia, and a new coronavirus strain was isolated from his lung tissue [1]. Another case of acute respiratory disease was diagnosed in a 49-year old male in London who was from Qatar and a new strain of coronavirus was isolated from this patient as well [2]. Shortly after, the entire genome of the new coronavirus was sequenced and deposited in the Genebank database under the number JX869059, KC164505.2. The phylogenetic analysis of the new virus genome revealed that homology of the nucleotide sequence between two cases was 99.5% and the isolates were closely related to bat coronavirus (Bat-CoV) which belongs to group 2C of β-coronavirus [3]. According to the recommendations by the International Committee on Taxonomy of Viruses (ICTV), the new coronavirus was named as ‘Middle East Respiratory Syndrome Coronavirus’ (MERS-CoV) [4]. Although, initially reported from the Middle East, MERS-CoV exported cases have also been observed worldwide.

With regard to viral origin and transmission, the first case of MERS-CoV infection did not relate it to any particular contact with animals before the disease onset; however, other studies did link it to Dromedary camels [5–8]. Beta-coronaviruses are strongly associated with bats serving as reservoir host, particularly, the African *Neoromicia* bats were speculated to be the natural reservoir of MERS-CoV [9–12]. Notably, serological evidence suggests that MERS-CoV has been in the circulation for at least 2–3 decades in dromedary camels [13]. Given that, the ancestral origin of MERS-CoV links it to African bats whereas, dromedary camels have been functioning as an intermediate host for this virus for a significantly long period of time [14–16]. Health facilities, hospitals and households with MERS patients are considered to be the epidemic centers of MERS-CoV outbreaks. MERS-CoV is mainly spreading across the geographical region of the Middle East while only sporadic cases are reported in the Europe, North America, Africa, and lately Asia. This may be because of the widespread population of *Dromedary* camels in the Middle East; however, the scientific proof of evidence that camel farms are a potential source of MERS-CoV infections still remains to be established. Besides, the typical seasonality pattern is not seen in case of MERS-CoV infections and only one report links it to camel breeding season. The modes of MERS-CoV transmission by droplet, contact, or airborne are not yet confirmed as well and thus its transmission among animals and from animals to human and human to human remains unclear. There is also no documentation available regarding MERS-CoV transmission during airplane flights. Therefore, standard infection prevention and control procedures are followed including droplet and airborne precautions. The viral incubation period is from 2 days to 2 weeks. The viral cytopathic effects clearly show prominent syncytium formation in humans as well as non-human primates. MERS-CoV targets directly the lower respiratory tract (pneumocytes) in dromedary camels and continues to replicate preferentially in the airway cells of the upper respiratory tract.

The clinical manifestations of MERS-CoV infections represent a wide spectrum ranging from asymptomatic cases to the ones with severe respiratory indexes. According to the WHO, MERS-CoV infection is an acute respiratory infection involving pyrexia of 38°C or more, cough with radiologic pulmonary presentation and also the history of the patients originating from or travel to the Arabian Peninsula and its neighboring countries within 10 days of symptoms. MERS-CoV cases have been identified as both community- and hospital-acquired, mainly among the aged population and in patients with multiple comorbidities such as acute pneumonia, upper respiratory tract infections, influenza-like illness, or asymptomatic infection(s) in children and immunocompromised hosts. Moreover, the common extra-pulmonary symptoms include diarrhea and acute renal failure. The early clinical diagnostic changes include the impaired liver and renal functions, lymphopenia, leukopenia and thrombocytopenia whereas leukocytosis, and neutrophilia are linked to progressive infections. The gold standard for diagnosis is detection of viral RNA by RT-PCR in compliance with the WHO guidelines for positive case criteria. The virus is found to be present in different diagnostic specimens such as the lower respiratory tract, sputum, endotracheal aspirate, bronchoalveolar lavage; upper respiratory tract, nasal or nasopharyngeal swabs, urine, feces, and blood. Nevertheless, positive biopsy and autopsy tissue specimens still remain to be reported.

Direct or indirect contact seems to explain a part of the transmission kinetics observed between dromedary camels and humans. In the general population, transmission is rather inefficient (R0<0.7) and it was reported that MERS-CoV mortality rate is 35% [17]. However, once the virus is introduced into hospital setting with large numbers of susceptible patients at risk, the virus appears to be transmitted very efficiently among such vulnerable host populations. Regarding viral evolution and natural reservoir, dromedary camels are the natural reservoir for MERS-CoV and given the multitude of different clades found in both dromedary camels and human outbreaks, it appears that virus evolution takes place in the reservoir host rather than in humans. The study objective was to report the prevalence of MERS-CoV infections in the GCC countries and to also investigate its demographics in the Arabian Peninsula.

## Methodology

The data for the last 4 years were collected form the King Abdulaziz medical city, Riyadh, KSA. Further data were collected from WHO S1 Table and also from ministry of health portals of the GCC countries as follows: Bahrain http://www.moh.gov.bh/, Kuwait www.moh.gov.kw, Oman www.moh.gov.om, Qatar www.moph.gov.qa, United Arab Emirates http://www.moh.gov.ae/, and Saudi Arabia http://www.moh.gov.sa/. We also consulted the GCC countries’ reports and websites for the incidence of MERS-CoV infections between June 2012 and July 2016. Furthermore, we searched PubMed database for articles form GCC countries reporting MERS-CoV infections.

Epidemiological data including age, sex, symptoms, date of onset, and date of sampling were collected and entered into Excel worksheets. Descriptive analysis, frequencies and percentages were calculated using SPSS vr. 20 statistical software.

## Results

### MERS-CoV within the time period from June 2012 to July 2016

**Fig 1:**
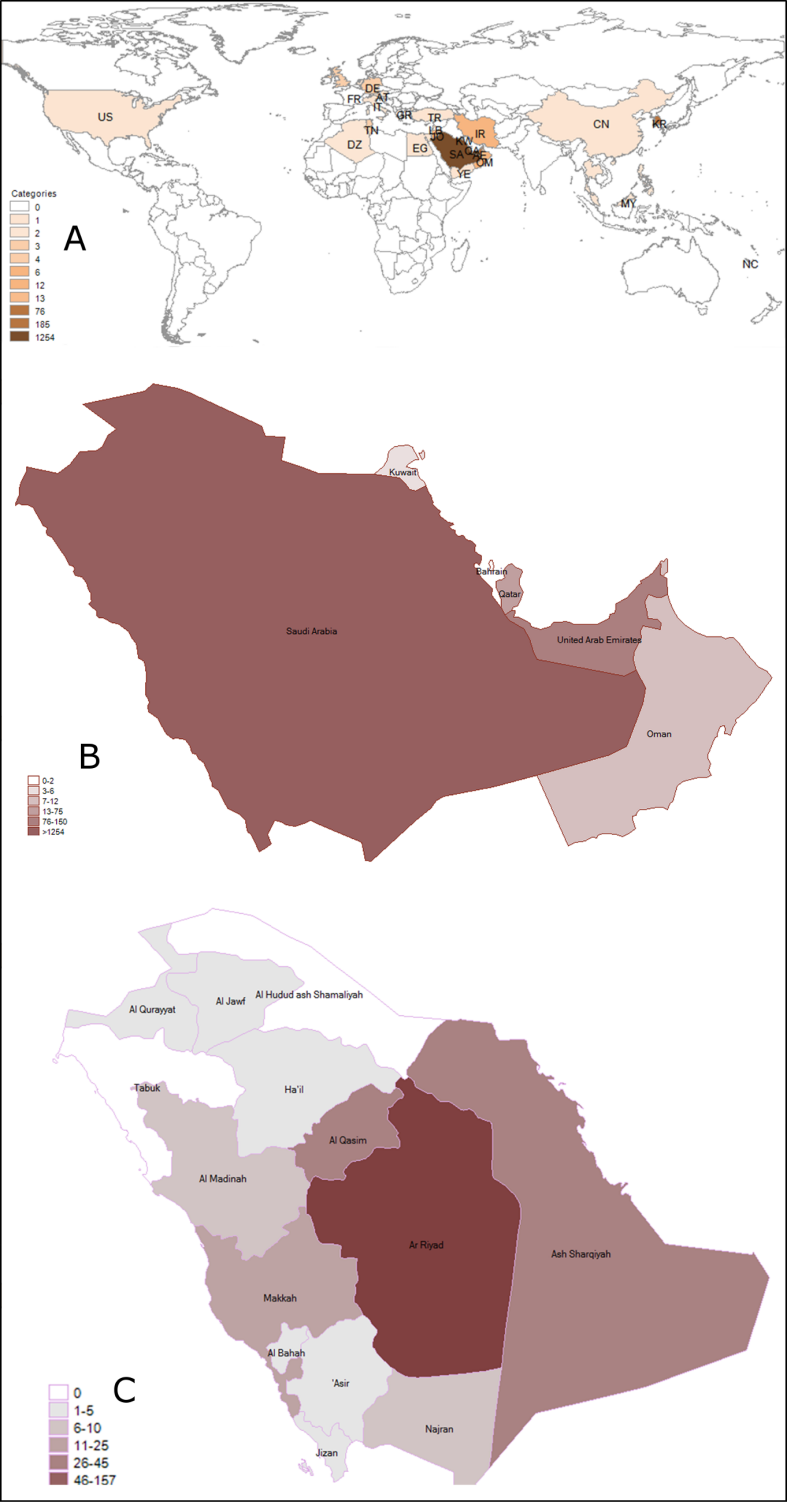
Magnitudes of positive MERS-CoV (A) worldwide, (B) among GCC countries 2012–2016 (C) Saudi Arabia by region.

Between June 2012 and July 2016, a total of 1797 confirmed MERS-CoV cases were reported worldwide with a mortality rate of 38.2% (n = 687). The regional distributions of MERS-CoV were as follows: Middle East had the highest number cases (88.4%), followed by Asia (10.7%), Europe (0.8%) and USA with only 2 cases officially reported (0.1%) The data are summarized in **Table 1** and **Fig 1A**.

**Table 1:** Distribution of MERS-CoV infections in humans by region and country, along with the date of first incidence and last reporting (Countries with the highest number of cases are highlighted in bold).

Region	# of Countries	Cases by region	Country	Cases by country	First incidence	Last reported
Middle East	13	1590	Lebanon	1	22/04/2014	22/04/2014
			Yemen	1	17/03/2014	17/03/2014
			Bahrain	1	10/04/2016	10/04/2016
			Egypt	1	22/04/2014	22/04/2014
			Algeria	2	23/05/2014	23/05/2014
			Tunisia	3	01/05/2013	17/06/2013
			Kuwait	4	30/10/2013	08/09/2015
			Iran	6	11/5/2014	18/03/2015
			Oman	8	26/10/2013	03/01/2016
			Qatar	16	15/08/2013	12/06/2016
			Jordan	26	02/04/2012	26/09/2015
			United Arab Emirates	80	19/03/2013	09/06/2016
			**Saudi Arabia**	**1,441**	**13/06/2012**	**29/07/2016**
Asia	5	198	China	1	21/05/2015	21/05/2015
			Malaysia	1	08/04/2014	08/04/2014
			Thailand	2	10/6/2015	14/01/2016
			Philippines	3	15/04/2014	30/06/2015
			**Korea**	**185**	**11/5/2015**	**02/07/2015**
Europe	8	14	Austria	1	22/09/2014	22/09/2014
			Turkey	1	25/09/2014	25/09/2014
			Italy	1	25/05/2013	25/05/2013
			Greece	1	08/4/2014	08/04/2014
			Germany	2	05/10/2012	07/03/2015
			Netherlands	2	01/05/2014	05/05/2014
			France	2	23/04/2013	27/04/2013
			**U.K.**	**4**	**03/09/2012**	**05/02/2013**
Americas	1	2	**USA**	**2**	**14/04/2014**	**1/5/2014**
	**Total**	**1797**				

### MERS-CoV occurrence according to exposure

One hundred fifty five patients out of the total 1797 confirmed cases (8.6%), reported their exposure to animals, of which, 130 out of 155 cases (83.9%) were exposed to camels, while 25 out of 155 (16.1%) stated exposure to other animals including sheep, cows and poultry (**Table 2**). Despite the fact that 674 out of 1797 MERS-CoV cases (37.5%) were health care-associated infections and 284 out of 1797 cases (15.8%) involved contact with an infected family member, 147 cases (8.2%) were still reported with no exposure to any of the above. The exposure data were found missing for the remaining 537 (29.9%) patients.

**Table 2:** Number of cases reported based on exposure. (Percentage (%) are shown in parentheses).

Animals			Healthcare-associated infection	Family member-associated infections	No exposure	Missing data	Total
Camel	Others	Total					
130/155	25/155	155	674	284	147	537	1797
(83.9)	(16.1)	(8.6)	(37.5)	(15.8)	(8.2)	(29.9)	(100)

### GCC demographics and MERS-CoV incidence

The distributions of MERS-CoV infections among 6 Gulf countries are illustrated in **Table 3** and **Fig 1B**. The majority of MERS-CoV infections (93%) reported between the time period from June 2012 to July 2016, were from Saudi Arabia. While, the remaining 5 GCC countries contributed only 7% of the cases with the distributions as follows: United Arab Emirates (5.0%), Qatar (1.0%), Oman (0.5%), Kuwait (0.2%) and Bahrain (0.06%). Gender analysis shown in **Table 3** reveals that 60% of the patients were male and 31% were female. Moreover, age-specific risk distributions of MERS-CoV showed a positive correlation between the incidence and the age. Regarding cases reported from Saudi Arabia, 2% occurred in age group ≤20 yrs while 25% cases were observed in patients aged 20–39 yrs. The highest risk age group was 40 yrs and above. Together, they represent 65% of all cases reported from Saudi Arabia.

**Table 3:** Gender- and age-specific distribution of MERS-CoV infections among the GCC countries.

Country	Total	By gender		By age				Missing data
		Male	Female	<20	20–39	40–59	≥60	
Bahrain	1	1	0	0	0	0	1	0
Kuwait	4	3	0	0	0	2	1	1
Oman	8	6	1	0	2	3	2	7
Qatar	16	10	1	0	1	3	6	6
**Saudi Arabia**	**1441**	**871**	**446**	**33**	**356**	**457**	**471**	**124**
UAE	80	56	22	3	32	27	16	2
**GCC total**	**1550**	**947**	**470**	**36**	**391**	**492**	**497**	**140**

### MERS-CoV infections by GCC country

#### Saudi Arabia

Next, we sought out the detailed demographic distributions of reported cases among 14 governorates in Saudi Arabia. As shown in **Table 4** and **Fig 1C**, most of the cases (52%) were reported in Al Riyadh region, making it the worst hit area in the country followed by Makkah (20%), Ash Sharqiyah (11%), Al Madinah (4%) and Najran (3%). The remaining 9 regions together contributed to 5% of the cases. To date, cases are still logged from KSA and newly-diagnosed positive cases are on the rise.

**Table 4:** Year wise distribution of MERS-CoV infections reported from various governorates of Saudi Arabia.

Region	2012	2013	2014	2015	2016	Total
Al Bahah	0	0	0	0	0	0
Al Jawf	0	2	9	2	1	14
Al Madinah	0	7	38	8	3	56
Ash Sharqiyah	0	30	25	67	34	156
Al Qasim	0	1	0	3	1	5
Al Qurayyat	0	0	1	1	0	2
**Ar Riyad**	**5**	**47**	**201**	**305**	**198**	**756**
'Asir	1	7	1	3	2	14
Ha'il	0	0	0	2	1	3
Makkah	0	8	258	32	67	365
Al Hudud ash Shamaliyah	0	0	7	1	0	8
Najran	0	1	11	17	8	37
Jizan	0	0	0	1	1	2
Tabuk	0	0	15	6	2	23
**Total**	**6**	**103**	**566**	**448**	**318**	**1441**

#### Bahrain

To date, there was only one case reported from Bahrain in Manama region. Herein, a 61-year-old Saudi male was admitted on the 29^th^ of March, 2016 to a health care facility in Bahrain for an unrelated medical condition. This person was later on tested as positive for MERS-CoV (**Table 1**).

#### State of Kuwait

According to Kuwait Ministry of Health, a total of 4 cases were confirmed as MERS-CoV infections. The first case was reported form the capital (Kuwait city) on October 2010, followed by 2 other reported cases, one each in 2013 and 2014. The last case, again reported from Kuwait city, was a 78-year-old male who developed symptom on the 8^th^ of September, 2014 and was tested positive for MERS-CoV infection.

#### State of Oman

Reports from both Oman ministry of Health and the WHO show that 8 patients were confirmed positive for MERS-CoV until now. The first case was a 68-year-old patient from Dakhliyah Governorate who was tested and found positive on October 29, 2013, with a history of no camel contact. Another case was reported in December 2013, involving 59-year-old male who had attended a camel race in the United Arab Emirates (UAE). Five more cases were reported during the time period from 2014 to 2015. The last case was reported from North Batinah Governorate which involved a 44-year-old male who developed symptoms on the 25^th^ of December, 2015. The patient was exposed to dromedary camels 14 days earlier to the onset of symptoms and was confirmed positive for MERS-CoV on January, the 23^rd^, 2016.

#### United Arab Emirates

The UAE among the GCC countries has the second highest number (80 cases) of MERS-CoV infections that occurred over the time period from March 2013 to June 2016. According to the UAE Ministry of Health and the WHO data, the first MERS-CoV confirmed case in the UAE involved an 82-year-old male and there were 21 reported outbreaks in UAE during 2013–2015, while 6 outbreaks in the year 2015 alone. The highest number was reported in 2014 while 5 small-scale outbreaks were documented in 2013. Three cases were reported in 2016. Among the latest cases reported in January 2016, a 73-year-old male and an 85-year-old female from Abu Dhabi were traced to another confirmed MERS-CoV patient with no history of exposure to camels or other risk factors. Finally, a 37-year-old expat male from Abu Dhabi developed symptoms on the 9^th^ of June, 2016 and was later tested positive for MERS-CoV.

### Seasonality of the occurrence of MERS-CoV infections

**Fig 2:**
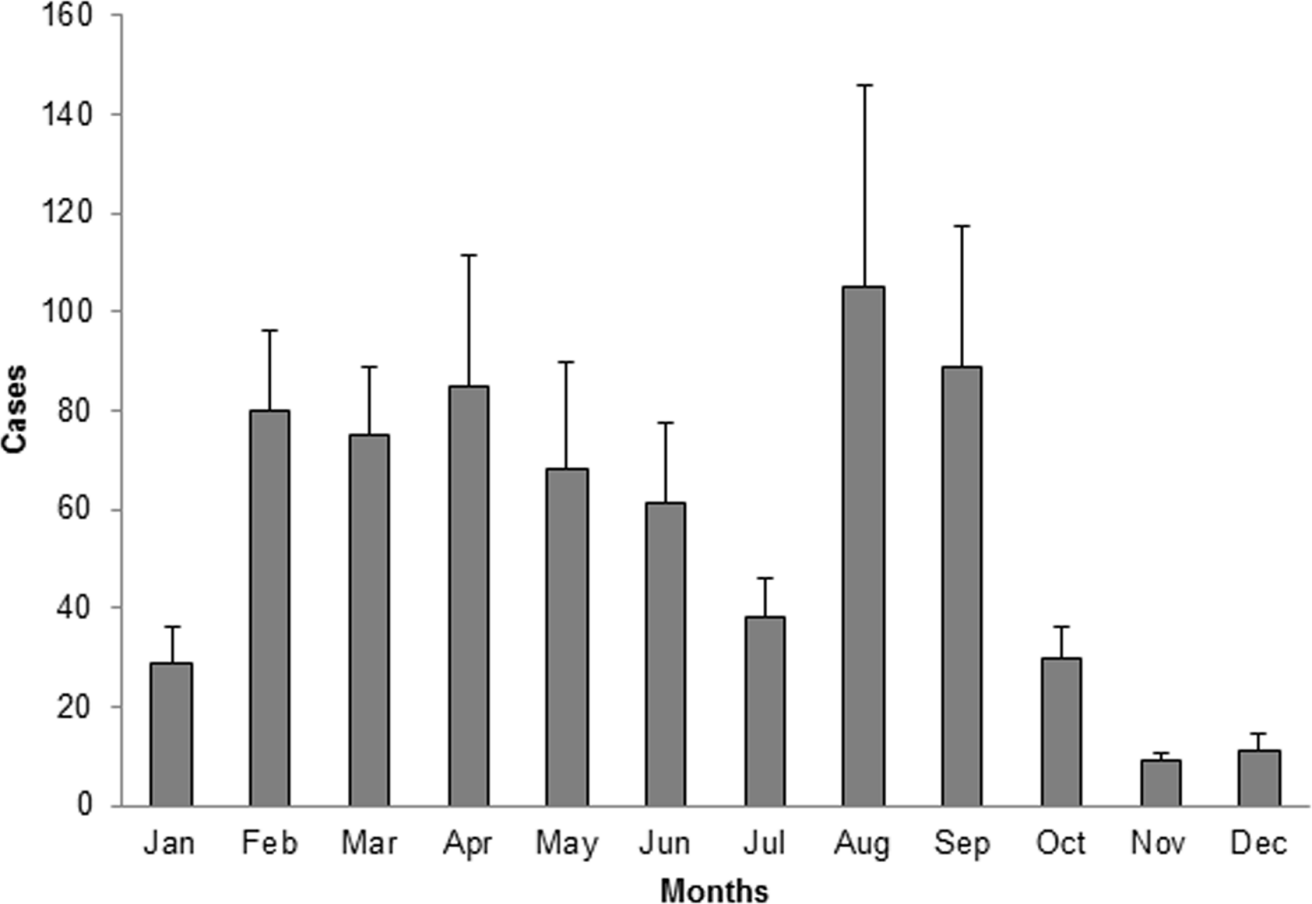
Seasonal pattern of MERS-CoV infections in Saudi Arabia from June 2012 to July 2016.

Finally, we searched for the pattern of MERS-CoV infections over the months in order to identify seasonality relationship. As shown in **Fig 2**, the average of the reported cases during this 4 years period shows that 60–80 cases are reported in the period between February and May, while about 90–100 are reported in August and September. In addition, a low point of infection occurs in the period of October—January and another one in June. The highest number of cases were reported overall during the summer time.

## Discussion

Over the past 4 years or so, increasing numbers of MERS-CoV infections have been reported from the Middle Eastern region [1]. Herein, we present a prevalence update on the current status of MERS-CoV infections in the GCC countries. The data collected over a time period from June 2012 and July 2016 show that the highest number of cases (1441) were reported from Saudi Arabia (93%) among a total of 1797 cases reported worldwide. Overall, a total of 1550 cases were reported only from the GCC region. The Saudi Arabian capital city of Riyadh with 756/1441 (52.4%) cases remained the hardest hit area for MERS-CoV outbreaks. The incidence of MERS-CoV infections was found to be highest among the elderly population aged 60 yrs or above. Moreover, the gender analysis showed that there were twice more number of males infected (871/1317) than females (446/1317). There is no evidence that MERS-CoV has gender predisposition[18]. The observed gender-related rates could be simply due to the higher probability of male exposure to camel population than females in the region[18, 19]. Furthermore, over one third (37.5%) of MERS-CoV patients received intensive care among all hospitalized cases [20]. One could argue that the hot climate shared by Arabian Peninsula and Sub-Saharan African region could contribute to the spread of MERS-CoV infections across these geographical regions. Although lesser in number, there are still numerous MERS-CoV infections recorded in Saudi Arabia during the winter time as compared with summer. The seasonality pattern analysis identifies a 2-phase annual cycle wherein the outbreaks occur during the winter and summer months. Altogether, the summer time represents the peak season for MERS-CoV infections and transmission.

The evolutionarily related bat virus might have undergone modifications and adaptation in order to be able to successfully infect and multiply in the camel as an intermediate host before transmission to the human host [21]. Although, camel is a well-known animal that is widely colonized in the Gulf region, it is also reared and maintained in other parts of the world. We speculate that there might be certain conditions or factors involved with regard to camel herding and shepherding exclusively in Saudi Arabia that would have facilitated and contributed to the survival of pathogen and fast spread of MERS-CoV infections from camels to humans across all over the country. There are some reports showed that human consumption of unpasteurized camel milk and or other camel products maybe a reason for the zoonotic transmission of MERS-CoV in the region[22–24]. On the other hand, there is strong evidence that MERS-CoV has been circulating in the dromedary camel population for more than 2 decades[25]. Yet, the reason that first human infected case was identified in 2012 remains unclear.

We found that mortality rates were higher among the elderly group for both genders which was also concordant with a previous study [26]. The possible explanation for the enhanced mortality in aged patients could be the presence of senescence-associated immune vulnerability in these individuals and suboptimal immune reactivity following a systemic challenge by exposure to MERS-CoV natural infection. Other Gulf countries show a few sporadic cases which may be due to the missing data that still have to be set straight or it could possibly be due to small size of camel populations is wide spread across vast geographical region. There may be still other factors involved that remain hidden at present but contribute significantly to the survival, transmission and pathogenesis of this relatively newly identified pathogen in this region of the world. Notably, it appears as if coronaviruses are able to cause serious viral infections when transferred from their reservoir (wild bat) host to the human host as observed previously for Ebola virus as well which was transmitted from wild bats to humans in Africa. Actually, many of MERS-CoV cases are initiated in rural areas and following hospitalization, further cases were reported. The majority of the MERS-CoV outbreak cases took place at health facilities; index cases are very crucial and they raise the question of the route of transmission of this zoonotic virus. This warrants caution that strict healthcare protocols and guidelines need to be followed and practiced by health care personnel in order to prevent the new outbreaks of MERS-CoV.

In conclusion, MERS-CoV infections were reported to occur in Saudi Arabia during the whole year whereas the incidence of human outbreaks peaked in winter and summer months. The disease incidence was also highest among the elderly population aged 60 yrs and above. Besides, the fact that majority of these cases were due to human to human interaction i.e. especially among the hospitalized ICU patients and not due to camel to human transmission, the local health sectors need to be made aware to mandate implementation of effective control strategies and stringent compliance with better standards of health and hygiene nationwide. Despite all whistle blowing efforts aimed at raising awareness of the magnitude of the problem at home, further efforts are still needed for proper treatment and care of MERS-CoV-infected patients in this country. Last but not least, the availability detailed reports of each and every case of MERS-CoV infection globally, and the GCC region particularly will provide valuable information to the scientific community that may be used to track, contain, and eradicate this disease more effectively.

## Supporting information

S1 Table MERS- CoV outbreak data and sources. (XLSX)
